# Effects of Angiotensin Receptor Blockers on Streptozotocin-Induced Diabetic Cataracts

**DOI:** 10.3390/jcm12206627

**Published:** 2023-10-19

**Authors:** Gaku Ishigooka, Hiroshi Mizuno, Shou Oosuka, Denan Jin, Shinji Takai, Teruyo Kida

**Affiliations:** 1 Department of Ophthalmology, Osaka Medical and Pharmaceutical University, Osaka 569-8686, Japan; hiroshi.mizuno@ompu.ac.jp (H.M.); shou.oosuka@ompu.ac.jp (S.O.);; 2 Department of Innovative Medicine, Osaka Medical and Pharmaceutical University, Osaka 569-8686, Japan; denan.jin@ompu.ac.jp (D.J.); shinji.takai@ompu.ac.jp (S.T.)

**Keywords:** diabetic cataract, candesartan, NOX-1, NOX-4, iNOS

## Abstract

This study aimed to determine the role of oxidative stress produced by the renin–angiotensin system (RAS) in cataract formation in streptozotocin-induced diabetic rats (STZ) using angiotensin II receptor blockers (ARBs). Rats were treated with streptozotocin and orally administered candesartan (2.5 mg/kg/day) or a normal diet for 10 weeks until sacrifice. Cataract progression was assessed through a slit-lamp examination. Animals were euthanized at 18 weeks, and the degree of cataract progression was evaluated. Oxidative stress was also assessed. In STZ-treated rats, lens opacity occurred at 12 weeks. Cataract progression was inhibited in the ARB-treated group compared with the placebo group (*p* < 0.05). STZ-treated rats exhibited upregulated angiotensin-converting enzyme (ACE) gene expression than control rats. Oxidative stress-related factors were upregulated in the placebo-treated group but suppressed in the ARB-treated group. A correlation coefficient test revealed a positive correlation between ACE gene expression and oxidative stress-related factors and a negative correlation between ACE and superoxide dismutase. Immunostaining revealed oxidative stress-related factors and advanced glycation end products in the lens cortex of the placebo-treated group. The mechanism of diabetic cataracts may be related to RAS, and the increase in focal ACE and angiotensin II in the lens promotes oxidative stress-related factor production.

## 1. Introduction

The global diabetes prevalence among individuals aged 20 to 79 years in 2021 was estimated at 10.5% (536.6 million individuals) and is projected to rise to 12.2% (783.2 million) by 2045 [1]. Hyperglycemia in diabetes mellitus causes diabetic cataracts, often accompanied by retinopathy, resulting in vision loss [2]. Surgical therapy is the standard treatment for diabetic cataracts [3]. However, as the number of patients with diabetes increases, less invasive and simpler treatment methods become ideal.

The formation of diabetic cataracts has been attributed to factors such as oxidative stress, osmotic stress, and an upregulated polyol pathway [4,5,6,7,8]. Recent narrative reviews have focused on oxidative stress [9], and many drugs and supplements have been investigated for their potential to suppress oxidative stress [10,11,12,13].

Hypoxia induces angiotensin-converting enzyme (ACE) production in diabetic retinopathy, leading to advanced glycation end products (AGEs) production via nicotinamide adenine dinucleotide phosphate (NADPH) in diabetic rats [14]. During this extensive study [14], the early onset and progression of diabetic cataracts were coincidentally observed. Thus, it was hypothesized that administering angiotensin receptor blocker (ARB), candesartan, might delay cataract progression in diabetic rats compared with controls.

The aqueous humor serves as a source of nourishment for the cornea and lens, both of which lack vascular supply in healthy eyes. Additionally, the renin–angiotensin system (RAS) is present in human aqueous humor [15]. The local RAS, operating through this aqueous humor circulation, is involved in cataract formation in angiotensin II-induced rats [16], and ARBs are effective in suppressing oxidative stress in diabetic cataracts [17]. Therefore, the RAS is hypothesized to participate in oxidative stress associated with the formation of diabetic cataracts. However, the underlying mechanism remains unknown.

In this study, we performed a series of experiments employing candesartan, an ARB that inhibits diabetic retinopathy, to elucidate this involvement [18]. We hypothesized that the RAS is similarly implicated in oxidative stress production during diabetic cataract production and potentially inhibits cataracts in streptozotocin-induced diabetic (STZ) rats, a well-known type 1 diabetes rat model. In clinical practice, ARBs are widely used to manage systemic hypertension in patients with diabetes [19], making our study potentially valuable as it suggests that ARBs can easily treat other diabetic complications. Here, we aimed to determine the role of oxidative stress produced by the RAS in cataract formation in STZ rats using ARBs. We focused on the RAS in diabetic cataracts and conducted kinetic analyses of the local RAS and oxidative stress-related factors in rats with diabetic cataracts. This study suggests that the pathological mechanism of diabetic cataracts may be related to an increase in oxidative stress factors through the enhancement of local angiotensin II action via increased ACE in the lens.

## 2. Materials and Methods

### 2.1. Animals

Twenty-eight, 7-week-old male Wistar rats weighing between 190 and 220 g were purchased from SLC Japan Inc. (Shizuoka, Japan) and housed in an air-conditioned room at approximately 23 °C with 60% humidity. They were maintained on a 12 h light/dark cycle and provided food and water ad libitum. Each rat was identified through an auricular procedure conducted under anesthesia at diabetes induction. The animals were handled following the ARVO Statement for the Use of Animals in Ophthalmic and Vision Research. Our experimental protocols conformed to the Animal Research: Reporting In Vivo Experiments (ARRIVE) guidelines [20] and were approved by the Osaka Medical and Pharmaceutical University Committee on the Use and Care of Animals (approval number: 2019-119).

### 2.2. Chemicals

All chemicals were obtained from Sigma-Aldrich (St. Louis, MO, USA) unless otherwise specified.

### 2.3. Anesthesia and Euthanasia

All procedures were performed under anesthesia using an intraperitoneal injection of medetomidine (0.75 mg/kg), midazolam (4 mg/kg), and butorphanol (5 mg/kg), as previously described. A slit-lamp examination was performed under isoflurane inhalation anesthesia. The rats were euthanized by exposure to CO_2_ in wood-shaving bedding.

### 2.4. Diabetes Induction

Diabetes was induced by administering a single 40 mg/kg intravenous injection of streptozotocin in 10 mM sodium citrate buffer (pH 4.5) to each rat after overnight fasting at 7 p.m. As a control, non-diabetic animals received an injection of citrate buffer only. Rats with blood glucose levels > 300 mg/dL 24 h after injection were considered diabetic. Rats with blood glucose levels < 300 mg/dL were re-administered candesartan the day after injection. Their weights and blood glucose levels were monitored the following day and every 2 weeks to ensure sustained hyperglycemia. Blood glucose and weight measurements were obtained at 7 p.m. on the day of the STZ injection and at 3 p.m. subsequently. On the day of euthanasia, blood glucose and weight measurements were obtained at 8 a.m. All experiments were conducted on 18-week-old rats after diabetes induction.

### 2.5. Candesartan Administration

The rats were categorized into three groups: healthy Wistar rats as controls (*n* = 9), STZ rats as placebo (*n* = 9), and ARB-treated rats (*n* = 10). In allocating animals into these groups, we ensured no significant differences in body weight and blood glucose levels.

The dose of candesartan was calculated at 2.5 mg/kg/day, using the average daily food intake of rats as 20 g. The candesartan-mixed diet was prepared by initially powdering the normal food and adding water and candesartan. The mixture was stirred and dried until solidification.

The ARB-treated rats were orally administered candesartan (2.5 mg/kg/day) from week 10 until sacrifice at week 18, whereas rats in the placebo and control groups were fed with the normal diet.

### 2.6. Estimation of Cataract Formation

The lens was promptly removed after euthanasia. The lenses were bluntly extracted by creating a split plane on the cornea with a razor blade while grasping the eye at the optic nerve with forceps. Lens opacity was estimated using slit-lamp examinations at 8, 12, and 17 weeks. After the lenses were extracted, we evaluated the severity of cataracts using a grading scale of 1–4 based on the degree of lens opacity.

### 2.7. Real-Time Reverse Transcription Polymerase Chain Reaction (RT-PCR)

Total ribonucleic acid (RNA) was extracted from tissues using the TRIzol reagent (Life Technologies, Rockville, MD, USA) and dissolved in RNase-free water (Takara Bio Inc., Otsu, Japan). Total RNA (2.5 μg) was transcribed into complementary deoxyribonucleic acid using Superscript VIRO software (Invitrogen, Carlsbad, CA, USA). The messenger RNA levels were measured via real-time RT-PCR using Stratagene Mx3000P (Agilent Technologies, San Francisco, CA, USA). Primers for real-time RT-PCR of ACE, superoxide dismutase (SOD), glutathione peroxidase (GPX), NADPH oxidase (NOX)-1, NOX-4, inducible nitric oxide synthase (iNOS), and glyceraldehyde 3-phosphate dehydrogenase (GAPDH) were designed by Roche Diagnostics (Tokyo, Japan). The primers were as follows: 5′-tctgcttccccaacaagact-3′ (forward) and 5′-aggaagccaggatgttggt-3′ (reverse) for ACE; 5′-agaaacatggcggtccag-3′ (forward) and 5′-acggacacattggccacac-3′ (reverse) for SOD; 5′-cctaaggcattcctggtatctg-3′ (forward) and 5′-caccrtcatggaaaaacctc-3′ (reverse) for GPX; 5′-ggcatccctttactgacct-3′ (forward) and 5′-tgctgctcgaatatgaatgg-3′ (reverse) for NOX-1; 5′-gaacccaagttccaagctca-3′ (forward) and 5′-gcacaaaggtccagaaatcc-3′ (reverse) for NOX-4; 5′-catcagctcctcccaggac-3′ (forward) and 5′-aggggaccctgaccatct-3′ (reverse) for iNOS; and 5′-aatgtatccgttgtggatctga-3′ (forward) and 5′-gcttcaccaccttcttgatgt-3′ (reverse) for GAPDH. Each messenger RNA level was normalized to that of GAPDH.

### 2.8. Statistical Analyses

The means and standard errors of the mean were calculated. All graphs were represented using the standard error of the mean. Fisher’s least significant difference was employed for RT-PCR to determine statistical significance. Unless stated otherwise, two-tailed Student’s *t*-tests were used. The level of significance was set at *p* < 0.05. The correlations were made using Pearson’s correlation coefficient. Graphs were created using Microsoft^®^ Excel for Mac ver 16.78 (23100802).

### 2.9. Histological Examination

For histological examination, the left eyeballs of rats in each group were harvested under anesthesia (100 mg/kg, i.p.), fixed in methanol Carnoy’s fixative overnight, and embedded in paraffin. Subsequently, these paraffin blocks were cut into 4 µm thick cross-sections using a microtome (LITORATOMU, REM-710, Yamato Koki Kogyo Ltd., Saitama, Japan). Hematoxylin and eosin (H&E) staining was performed on the first section of each group using standard staining protocols. The remaining sections were subjected to immunohistological staining to identify the localization of NOX-1 (1:50 dilution, ab131088, Abcam, Cambridge, UK), NOX-4 (1:50 dilution, ab154244, Abcam, UK), iNOS (1:100 dilution, GTX130246, GeneTex, Irvine, CA, USA), carboxyethyl lysine (CEL) (1:50 dilution, AGE-M02, Cosmo Bio Co., Ltd., Tokyo, Japan), carboxymethyl lysine (CML) (1:50 dilution, KH011-01, Transgenic, Fukuoka, Japan), and ACE (1:100 dilution, Human ACE/CD143 antibody, RD systems Inc., Minneapolis, MN, USA). Immunostaining with the abovementioned antibodies was performed as previously described [5]. Briefly, deparaffinized sections were incubated with the respective antibodies overnight at 4 °C, followed by a reaction with components from a labeled streptavidin–biotin peroxidase kit (Dako LSAB kit; Dako, Carpinteria, CA, USA). Subsequently, the sections were incubated with 3-amino-9-ethylcarbazole for color development, counterstained with hematoxylin, and mounted on cover glasses. Slides were examined using an upright microscope (ECLIPSE Ni, Nikon, Japan).

## 3. Results

### 3.1. Effects of ARBs on Body Weight and Blood Glucose Levels in STZ Rats

**Figure 1:**
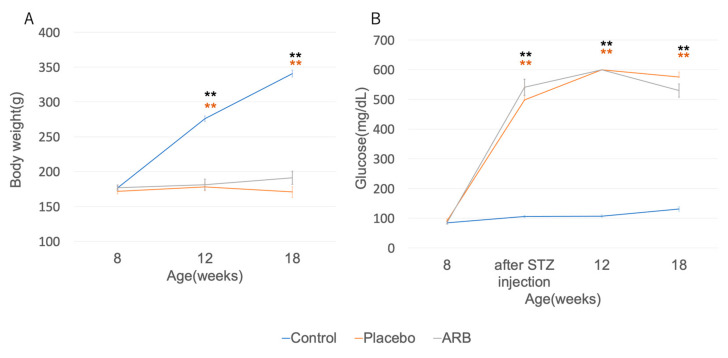
Body weight and blood glucose levels were measured at 8, 12, and 18 weeks of age, and blood glucose was measured the day after Streptozotocin (STZ) injection. (**A**) The control group (blue) exhibited increased mean body weight than the placebo group (orange) and the ARB group (black);s; (**B**) The placebo and ARB groups had increased mean blood glucose levels than the control group respectively (** *p* < 0.01). All STZ rats at 12 weeks showed blood glucose levels exceeding the measured range. No significant differences were observed in body weight and blood glucose levels between all groups at the beginning of the experiment (8 weeks). Furthermore, no significant differences were observed in body weight or blood glucose levels in STZ rats with or without ARB at any time point. ARB, angiotensin II receptor blocker.

The blood glucose level in STZ rats at the time of sacrifice was 416 ± 207 mg/dL. The control group had a higher mean body weight than the STZ group. In contrast, the blood glucose levels did not increase in the control group (Figure 1A). STZ rats showed significantly lower weight gain and elevated blood glucose levels than control rats (Figure 1B). No changes in body weight and blood glucose level were observed after administering ARB.

### 3.2. Cataract Progression in STZ Rats and Its Suppression by ARB

**Figure 2:**
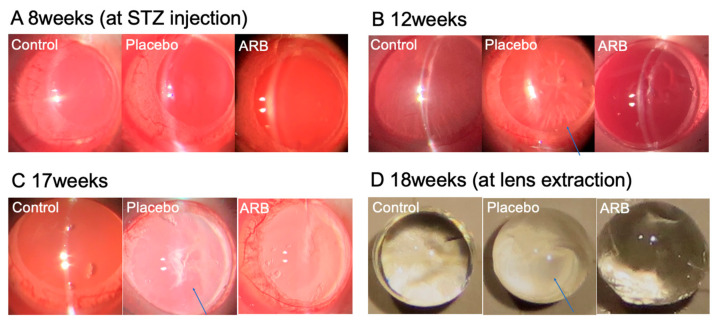
The lens of a rat was photographed during slit examination under inhalation anesthesia. (**A**) During Streptozotocin (STZ) injection, lenses were clear in all groups. (**B**) At 12 weeks, lens opacity (arrows) appeared in the placebo groups. (**C**,**D**) The opacity progressed at 17 weeks and during lens extraction. ARB, angiotensin II receptor blocker.

We observed the progression of lens opacity over time, which was most pronounced in the cortical zone of the lenses in the placebo group (Figure 2).

**Figure 3:**
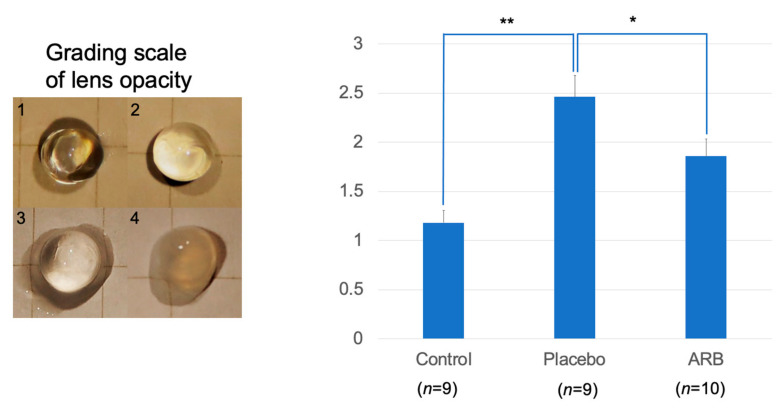
Lenses were extracted and rated on a scale of 1–4. Cataract formation significantly progressed in the placebo group (** *p* < 0.01). The progression was suppressed in the angiotensin II receptor blocker (ARB) group (* *p* < 0.05).

Cataracts were rated on a scale of 1–4 based on the degree of lens opacity at the time of extraction. Cataract formation significantly progressed in the placebo group (*p* < 0.01). The progression was suppressed in the ARB groups (*p* < 0.05) (Figure 3).

### 3.3. Cell Proliferation and Vacuolation in the Cortical Part of the Lens in H&E Staining

**Figure 4:**
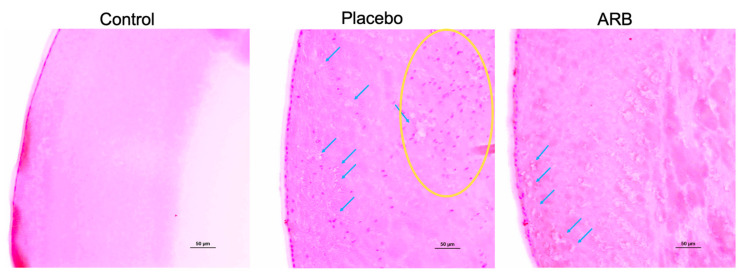
Hematoxylin–eosin staining in the lens cortex. Hyperplasia of lens cells (yellow circle) is evident in the lens cortex of STZ rats. Vacuolations of lens fibers are observed in the STZ rats (blue arrows). ARB, angiotensin II receptor blocker.

Hyperplasia of lens epithelial cells and degenerative lesions such as vacuolation of lens fibers were observed in the placebo group and the lenses of the STZ rats, and these findings were suppressed in the ARB-treated group. H&E staining showed that these factors were suppressed in the ARB group (Figure 4).

### 3.4. Inhibition of Oxidative Stress-Related Factors by ARBs and Changes in Antioxidant Enzymes in RT-PCR

The RT-PCR results revealed that ACE significantly increased in the STZ placebo group, but no significant difference was observed between the STZ groups.

**Figure 5:**
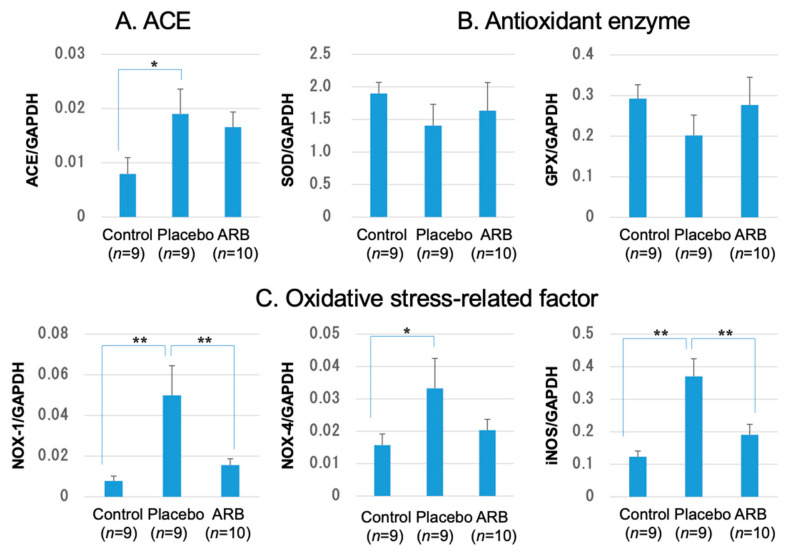
Results of real-time polymerase chain reaction: (**A**) Angiotensin-converting enzyme (ACE) significantly increased in the streptozotocin (STZ) placebo group. This increase was not suppressed in the angiotensin II receptor blocker (ARB) treated groups (* *p* < 0.05). (**B**) Superoxide dismutase (SOD) and glutathione peroxidase (GPX) showed a decreasing trend in the STZ placebo and an increasing trend in the ARB, but these trends were insignificant; (**C**) NADPH oxidase (NOX)-1 and inducible nitric oxide synthase (iNOS) significantly increased in the placebo group but significantly decreased in the ARB group. NOX-4 significantly increased in the placebo group and tended to decrease in the ARB group (* *p* < 0.05, ** *p* < 0.01). GAPDH, glyceraldehyde 3-phosphate dehydrogenase.

Antioxidant enzymes, including SOD and GPX, showed a decreasing trend in the STZ placebo group and an increasing trend in the ARB-treated group; however, these differences were insignificant (Figure 5).

**Figure 6:**
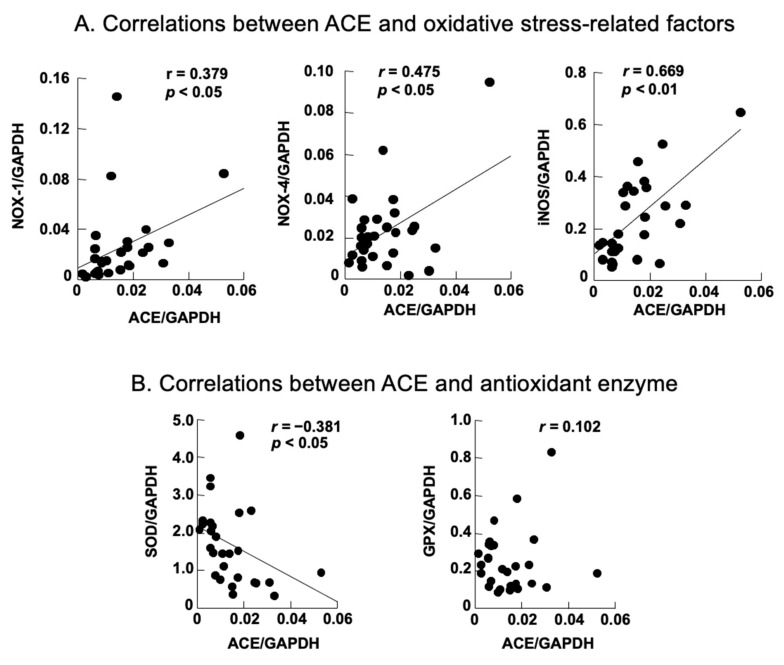
Correlation analysis based on reverse transcription polymerase chain reaction. (**A**) Angiotensin-converting enzyme (ACE) had a positive correlation with NADPH oxidase (NOX)-1 and NOX-4, especially with inducible nitric oxide synthase (iNOS); (**B**) ACE exhibited a negative correlation with superoxide dismutase (SOD), whereas glutathione peroxidase (GPX) did not exhibit a significant correlation. GAPDH, glyceraldehyde 3-phosphate dehydrogenase.

The levels of oxidative stressors, including NOX-1, NOX-4, and iNOS, were significantly elevated in the placebo group and significantly decreased in the ARB-treated group. ACE and oxidative stress-related factors showed a positive correlation, with iNOS demonstrating a particularly strong positive correlation. In contrast, ACE and SOD levels exhibited a negative correlation (Figure 6).

### 3.5. Suppression of Oxidative Stress-Related Factors and AGEs by ARBs in Immunostaining

**Figure 7:**
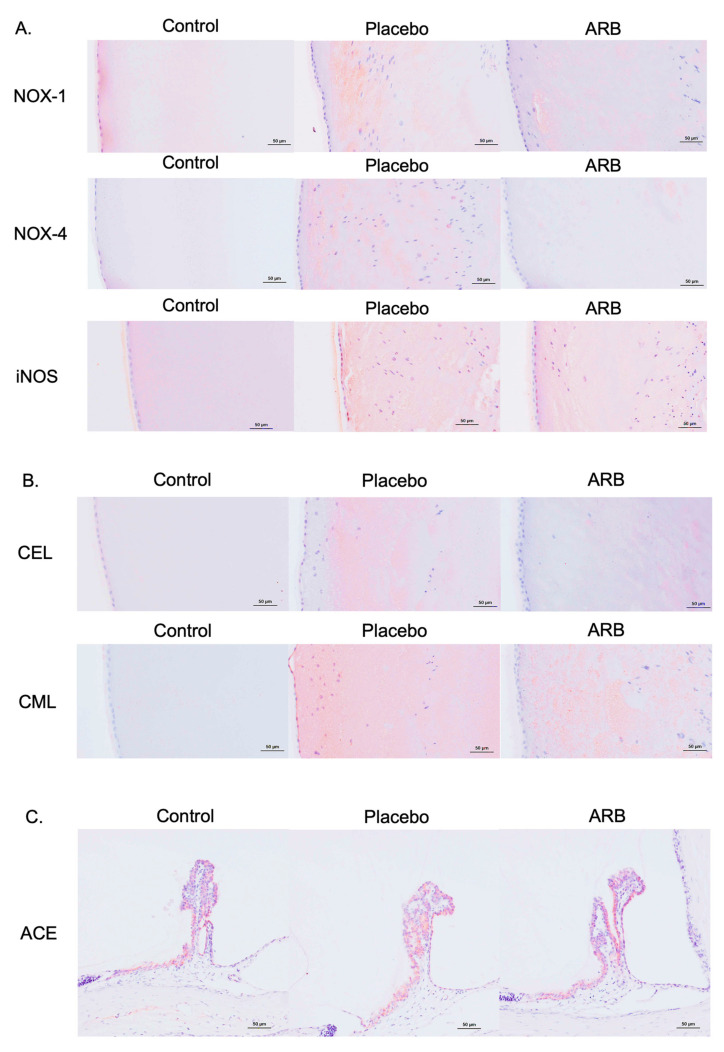
(**A**) Immunostaining of NADPH oxidase (NOX)-1, NOX-4, and inducible nitric oxide synthase (iNOS). (**B**) Immunostaining of carboxyethyl lysine (CEL) and carboxymethyl lysine (CML). (**C**) Immunostaining of angiotensin-converting enzyme (ACE) in the iris and ciliary body of the eye.

Immunostaining revealed the presence of oxidative stress-related factors, including NOX-1, NOX-4, and iNOS, in the lens cortex of the placebo group, whereas these factors were suppressed in the ARB-treated group (Figure 7A). In the placebo group, staining for AGEs, such as CEL and CML, was observed in the lens cortex. These factors and oxidative stress-related factors were suppressed in the ARB group (Figure 7B). ACE has been reported to be produced in the ciliary epithelium of rats in a previous study [6]. Therefore, we performed immunostaining for ACE in the ciliary body to clarify its localization in the eye. Positive staining for ACE was also observed in the ciliary body of all groups (Figure 7C).

## 4. Discussion

In this study, we discovered a significant correlation between ACE and oxidative stress-related factors through kinetic analyses of the RAS in rats with diabetic cataracts.

Chronic hyperglycemia in the retina is associated with elevated levels of oxidative stress produced by retinal cells, including glial and endothelial cells [21,22,23,24]. Moreover, RAS is locally activated in eyes with diabetic retinopathy [25], and its contribution includes the promotion of retinal neovascularization, inflammation, oxidative stress, and neuronal and glial dysfunction [26,27]. Moreover, candesartan, an RAS blocker, reduced the incidence of diabetic retinopathy [28,29,30]. Our colleagues have previously reported that hypoxia induces ACE production in diabetic retinopathy, leading to AGE production via NADPH [14]. Therefore, we hypothesized that in diabetic cataracts, an increase in ACE might increase the local RAS system through aqueous humor circulation, resulting in oxidative stress. Additionally, a study on RAS suppression of iNOS [31] and the progression of hypertensive cataracts in hypertensive rats [32] suggested that similar studies could be conducted in STZ rats, which are diabetic rat models. In this study, we investigated the effects of RAS on diabetic cataracts and ACE localization in the ciliary body.

We also observed that lens opacity occurred at 12 weeks in STZ rats, and cataracts occurred at 18 weeks. The progression of diabetic cataracts occurred significantly in the placebo group compared with the control group of similarly aged rats. However, the progression of diabetic cataracts was suppressed in the ARB-treated group. Our lens analyses across these three groups revealed that ACE levels significantly increased in the placebo group. Additionally, NOX-1, NOX-4, and iNOS levels significantly increased in the placebo group and significantly decreased in the ARB-treated group. In contrast, the opposite trend was observed for antioxidant enzymes. Positive correlations were observed between ACE and NOX-1, NOX-4, and iNOS, whereas a negative correlation was observed between ACE and SOD. Immunostaining experiments indicated positive staining for NOX-1, NOX-4, iNOS, CEL, and CML in the lens cortex of the placebo group. In this study, we suggested that the mechanism of diabetic cataracts may be deeply related to RAS, the increase in focal ACE and angiotensin II levels in the lens, and the promotion of oxidative stress factors, including NOX-1, NOX-4, and iNOS. Recent studies have revealed that the RAS and its oxidative stress products are involved in the formation of diabetic cataracts [10,17]; however, the mechanism is unclear. We demonstrated the inhibitory effects of ARBs on cataract progression. Histologically, suppression of degeneration in the cortical part of the lens was confirmed, suggesting an observable effect of oral administration. However, H&E staining did not reveal complete inhibition of degeneration; therefore, the effects might be limited to the inhibition of progression.

The RT-PCR results revealed that ACE, NOX-1, NOX-4, and iNOS increased in the lenses of STZ rats, and the positive correlation between ACE and NOX-1, iNOS, and NOX-4 indicated that ACE-induced enhancement of the RAS led to increased NADPH-mediated oxidative stress production in diabetic cataracts. The negative correlation between ACE and SOD levels may suggest an increase in oxidative stress, although no significant difference was observed in SOD levels between the groups in this study. The recovery trend in SOD and GPX levels in the ARB group can be attributed to the lack of significant difference and the trend, as this change is caused by suppressing consumption secondary to reduced reactive oxygen species production by ARBs. Therefore, the increase in ACE and RAS-mediated production of reactive oxygen species does not directly affect SOD and GPX but only indirectly consumes them. However, we must also consider that the number of rats was too small. Overproduction of oxidative stress causes an increase in AGEs [23]. In diabetic cataracts, staining of CML and CEL, the main structures of AGEs, was observed in the cortical zone of the lens. Cortical cataracts in slit examinations are derived from cortical opacities caused by AGEs.

Only ACE production in the iris and not in the aqueous humor was examined in this study; however, increased ACE production in the iris and aqueous humor might lead to increased ACE in the lens cortex, which is close to the anterior aqueous humor, and the accompanying increase in RAS, resulting in opacity in the cortical area. This is supported by the fact that proliferative diabetic retinopathy in humans increases ACE production in the aqueous humor [33].

Recently, various medicines and supplements aimed at suppressing diabetic cataracts, such as sodium-glucose cotransporter-2 inhibitors, vitamin C, vitamin K, and extracts, have been studied [8,10,12,13,34,35,36,37,38]. These preventive mechanisms are diverse and include inhibition of the polyol pathway as an inhibitory effect on reactive oxygen species [11], direct antioxidant effects [13], inhibition of overexpression of AGE receptors in lens epithelial cells via glucose transporter overexpression [10], and inhibition of lens aldose reductase 2 [12]. In addition to its known cardioprotective and renoprotective effects [39], candesartan has recently shown promise in suppressing neuronal apoptosis [40] and improving insulin resistance and hepatosteatosis by reducing intracellular calcium overload and lipid accumulation [41], which may alleviate various symptoms of diabetic complications. The results of this study may help demonstrate the benefits of candesartan.

This study had some limitations. No significant differences were observed in weight and blood glucose changes; however, a slight trend was observed toward improvement in the ARB group, suggesting the need to consider the systemic effects of ARBs. Moreover, we did not measure blood pressure in this study; thus, we did not investigate the potential improvement in systemic hypertension.

Furthermore, there may be some errors in the actual dosage of candesartan because it was administered through free feeding rather than force-feeding. A previous study administered losartan topically using eye drops and demonstrated a higher effectiveness. This result suggests that the topical administration of losartan is more effective than the ophthalmic administration [17], which should have been considered in this study. The study also examined changes in aldose reductase and various ionic activities, which were not investigated in this study and will be the subject of future work [17]. These fluctuations have also been observed with ARBs and should be examined in future studies.

No significant difference was observed in NOX-4 between the sham and ARB groups, and no significant difference was observed in antioxidant enzyme levels between these groups. Hence, future studies should increase the number of rats and utilize force-feeding. The specific mechanism of the correlation between ACE and NADPH has not been investigated; thus, in vitro studies should be conducted. Furthermore, although we confirmed ACE localization in the iris and ciliary body of the eyeballs, quantifying ACE production in the ciliary body alone may be challenging because of its small size. Whether ACE production in the iris is increased in STZ rats remains unclear, and the effect of ACE production on the lens via the anterior chamber fluid remains speculative.

Furthermore, future investigations should examine mutations in crystallin, which were not investigated in this study. Cataracts are caused by the deposition of aggregated proteins in the eye, leading to lens opacity, light scattering, and visual impairment [42,43]. α-crystallin are small heat shock proteins that play a central role in maintaining lens transparency and refractive properties. These proteins possess chaperone-like activity, effectively trapping denatured proteins. Moreover, α-crystallin protects cells from stress-induced apoptosis, regulates cell proliferation, and enhances genomic stability [43]. Mutational suppression of α-crystallin is also often observed when describing the inhibitory effect of diabetic cataracts [8,44,45,46]. In STZ rats, a decrease in the chaperone-like activity of α-crystallin was observed. The hyperglycemia-induced decrease in chaperone-like activity was associated with reduced hydrophobicity and changes in the secondary and tertiary structure of α-crystallin [47].

Furthermore, oxidative stressors in diabetes and reactive carbonyl compounds associated with diabetes have been demonstrated to alter the secondary to quaternary structure of human αA-crystallin. The formation of high molecular weight aggregates of these αA-crystallin species was significantly prevented by ascorbic acid and the antioxidant enzyme glutathione. The results of this study suggest the formation of the high molecular weight aggregates of these αA-crystallin [48].

The administration of candesartan might also suppress the denaturation of α-crystallin by suppressing the production of oxidative stress. However, quantification of insoluble proteins is required to verify this hypothesis.

Future tissue studies should investigate hypoxic conditions using cultured lens epithelial cells. The accumulation of AGEs in the cytoplasm of lens epithelial cells (LECs) and the high expression of iNOS have been implicated in the apoptosis of LEC changes associated with diabetic cataracts [49], and similar results can be expected with ARBs. Furthermore, we examined the results in Spontaneously Diabetic Torii rats, a model of type 2 diabetes.

## 5. Conclusions

This study suggests that the pathological mechanism of diabetic cataracts may be related to an increase in oxidative stress factors, including NOX-1, NOX-4, and iNOS, through the enhancement of local angiotensin II action via increased ACE in the lens. Early administration of ARBs may delay the progression of diabetic cataracts. Further studies will be conducted considering the number of rats, experimental models, and in vitro experiments. We had an oral presentation of this research at the ARVO 2023 meeting in New Orleans, USA, on 25 April 2023.

## Data Availability

Data are available in a publicly accessible repository. The data presented in this study are openly available in FigShare at https://doi.org/10.6084/m9.figshare.24314872.v2.
